# Comparative effects of dietary zinc nanoparticle and conventional zinc supplementation on broiler chickens: A meta-analysis

**DOI:** 10.14202/vetworld.2024.1733-1747

**Published:** 2024-08-04

**Authors:** Cecep Hidayat, Sadarman Sadarman, Danung Nur Adli, Ridho Kurniawan Rusli, Bachtar Bakrie, Simon Petrus Ginting, Santiananda Arta Asmarasari, Bram Brahmantiyo, Arif Darmawan, Hasnelly Zainal, Achmad Fanindi, Supardi Rusdiana, Iwan Herdiawan, Endang Sutedi, Yulianri Rizki Yanza, Anuraga Jayanegara

**Affiliations:** 1 Research Center for Animal Husbandry, Research Organization for Agriculture and Food, National Research and Innovation Agency of Indonesia, Cibinong Science Center, Jalan Raya Jakarta-Bogor, Cibinong, Bogor 16915, West Java, Indonesia; 2 Department of Animal Science, Faculty of Agriculture and Animal Science, Universitas Islam Negeri Sultan Syarif Kasim Riau, Pekanbaru 28293, Indonesia; 3 Animal Feed and Nutrition Modelling Research Group, Faculty of Animal Science, IPB University, Bogor 16680, Indonesia; 4 Department of Feed and Animal Nutrition, Faculty of Animal Science, Universitas Brawijaya, Malang, Indonesia; 5 Department of Nutrition and Feed Technology, Faculty of Animal Science, Universitas Andalas, Padang, 25175, Indonesia; 6 Department of Nutrition and Feed Technology, Faculty of Animal Science, IPB University, Bogor 16680, Indonesia; 7 Department of Animal Nutrition and Feed Technology, Faculty of Animal Husbandry, Padjadjaran University, Jl. Raya Bandung Sumedang KM 21, Jatinangor, Sumedang, 45363, Indonesia

**Keywords:** broiler chicken, conventional zinc, performance, welfare indices, zinc nanoparticles

## Abstract

**Background and Aim:**

Zinc (Zn) is important for various physiological processes in broiler chickens, including protein and carbohydrate metabolism, growth, and reproduction. The gastrointestinal absorption of Zn in broiler chickens was notably low. One approach that has been explored for enhancing the bioavailability of Zn is the development of Zn nanoparticles (NPs). Zn is required for various physiological processes in broiler chickens, including protein and carbohydrate metabolism, growth, and reproduction. Therefore, this study aimed to assess the impact of conventional Zn and Zn NPs on broiler chickens using a meta-analysis methodology.

**Materials and Methods:**

A database was built from published literature to evaluate the effects of the addition of Zn NPs and conventional Zn on broiler chicken responses, including the following parameters: production performance; carcass cuts; visceral organ weight; lymphoid organ weight; nutrient digestibility; intestinal villi; mineral Zn, calcium, and phosphorus concentrations; hematology; blood parameters; immunoglobulin; and intestinal bacterial population. Various scientific platforms, including Scopus, Web of Science, PubMed Central, and Google Scholar, were used to search for peer-reviewed articles. A database was created from 25 studies that met the inclusion criteria. The data were then processed for a meta-analysis using a mixed-model methodology. Different types of Zn (NPs versus conventional) were considered fixed effects, different studies were treated as random effects, and p-values were used as model statistics.

**Results:**

Across the parameters observed in this study, the use of Zn NPs was more efficient in Zn utilization than conventional Zn, as evidenced by the average dose of Zn NPs being much lower than that of conventional Zn (79.44 vs. 242.76 mg/kg) yet providing similar (p > 0.05) or even significantly better effects (p < 0.05) compared to conventional Zn usage.

**Conclusion:**

This investigation revealed the beneficial influence of Zn NPs in broiler chickens compared to the conventional utilization of Zn through an all-encompassing meta-analysis. Moreover, Zn NPs have proven to be more effective in Zn utilization when juxtaposed with conventional Zn, as demonstrated by the significantly lower quantity of Zn NPs administered compared to conventional Zn, while yielding comparable or even superior outcomes compared to the traditional utilization of Zn. A limitation of this study is that the Zn NPs used were sourced from inorganic Zn NPs. Therefore, future research should focus on evaluating the efficiency of organic Zn NPs in broiler chicken feed.

## Introduction

Broiler chicken farming is an industry with rapid growth and great interest in research and development focused on improving productivity, health, and disease resistance through feed utilization [1]. Feed represents the largest input cost of livestock production systems, including poultry farming. Research on animal feed continues to improve feed utilization efficiency with the aim of achieving production efficiency. One research direction in the field of animal feed is the application of nanotechnology.

Nanotechnology has been widely discussed owing to its numerous benefits that can be exploited across various disciplines [2]. Nanoparticles (NPs) have been increasingly used in chicken feed to enhance absorption and feed utilization efficiency [3]. NPs are smaller than 100 nm [4]. The application of nanotechnology to chicken feed is often related to the use of nanominerals. Zn is an essential mineral for livestock production. Zinc (Zn) is a vital mineral in poultry, as it plays a critical role in protein and carbohydrate metabolism, growth, and reproduction [5]. Inorganic forms of Zn, such as Zn oxide, Zn sulfate, and Zn chloride, and organic forms, such as Zn proteinases, Zn amino acids, Zn picolinate, and Zn methionine, are commonly used as feed additives or supplements in poultry rations [6]. It has been reported that Zn absorption in the digestive tract of broiler chickens is exceptionally low [7]. Recently, attempts have been made to enhance the bioavailability of Zn by developing Zn NPs [7]. Zn in NPs exhibits greater chemical reactivity than its conventional counterpart and participates in oxidation reactions with diverse organic compounds [8]. In addition, nanominerals have the ability to pass through the small intestine and enter the bloodstream as well as various organs, including the brain, lungs, heart, kidneys, spleen, liver, intestines, and stomach [4]. Zn in the NP form can enhance growth and immunity and act as an antibacterial agent [9].

Several studies on the effects of Zn, whether conventional or Zn NPs, in broiler chickens have revealed several factors, including research location, Zn source, chicken age, and chicken strain [5–7]. These variables may play a role in determining the production performance, immune response, and other parameters.

Based on these findings, a systematic review is required to determine the influence of the type of Zn used (conventional Zn vs. Zn NPs) on broiler chickens, and a strong conclusion. This meta-analysis evaluated the effects of conventional Zn and Zn NPs on broiler chickens.

## Materials and Methods

### Ethical approval

Peer-reviewed publications were carefully selected and assessed according to the protocols established by the PRISMA 2020 (https://www.prisma-statement.org/).

### Study period and location

This meta-analysis study was conducted from November 2023 to February 2024 at the Research Center for Animal Husbandry in Bogor, Indonesia. Communication and collaboration with all authors outside Bogor, Indonesia, were carried out online.

### Literature search strategy

Raw data were collected from selected articles that focused on the use of Zn NPs and conventional Zn in broiler chickens. Various scientific platforms, including Scopus (https://www.sciencedirect.com/), Web of Science (https://mjl.clarivate.com/search-results), PubMed Central (https://pubmed.ncbi.nlm.nih.gov/), and Google Scholar (https://scholar.google.com/), were used to search for peer-reviewed articles. Browser keyword generation adhered to the Population, Intervention, Comparison, and Outcome principle, defined as follows: P = “broiler AND chickens,” I = “nano OR zinc AND particles,” C = “control versus treatment (addition of nano zinc OR nano zinc particles),” and O = “growth AND performance (e.g., “nutrient digestibility,” “serum AND biochemical,” and “intestinal AND carcass quality”) [10].” The review period was from 2013 to 2023. In addition, the reference lists of each examined article were scrutinized to identify potentially relevant articles that might have been overlooked during the initial search.

### Eligibility criteria

The literature collected a total of 142 articles, which were then selected using the following criteria: (1) Written entirely in English, available in full text, and reports on the utilization of Zn from any source in broiler chickens of any breed and age; (2) the source of Zn used; (3) the dose of Zn used; and (4) a comparison of conventional Zn and Zn NPs. A total of 45 titles and abstracts were selected. Ninety-seven kinds of literature were excluded because their titles and contents were irrelevant to the observed topic. Twenty studies were excluded for several reasons (uninteresting comparisons, irrelevant parameters, and insufficient data for analysis).

### Article selections

**Figure-1:**
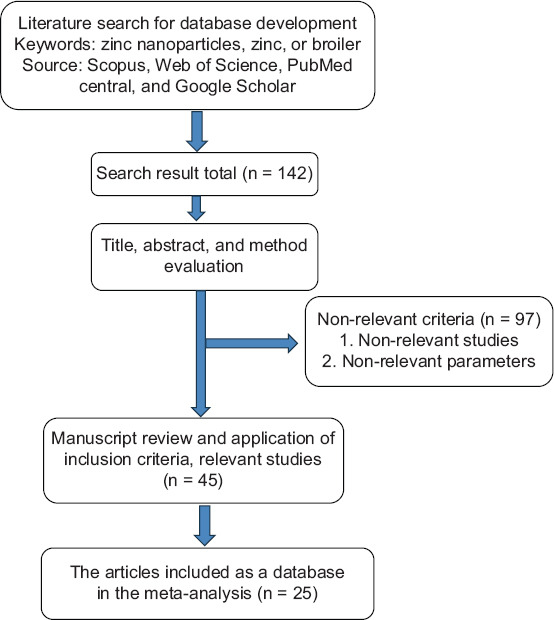
PRISMA flow chart for study selection in this meta-analysis.

The initial evaluation included 142 peer-reviewed publications that investigated the use of nano Zn particles and conventional Zn in broiler chickens. Of these, 97 publications were deemed irrelevant because of non-relevant parameters or studies, and 45 publications featuring non-relevant content or undergoing manuscript review were excluded. After a thorough examination of the full texts, a final total of 25 eligible papers were identified. The process for selecting studies for this meta-analysis is depicted in Figure-1, and a summary of the resulting dataset is provided in Table-1 [11–35]. After importing the articles from the scientific databases, four authors screened the titles and abstract lists, and three researchers determined the final papers for inclusion. The exclusion criteria were review articles, theses, dissertations, conference proceedings, book chapters, *in vitro* studies, and articles not written in English were excluded. Selected articles were stored in the Mendeley Library. Subsequently, detailed information and data from these articles were tabulated in a spreadsheet, including references, year, level administered, type of herbal plant extract, country, strain of broiler chickens, experimental duration, and outcomes. Graphical data and relevant figures from peer-reviewed published articles were extracted and converted using WebPlot Digitiser version 4.4 (https://automeris.io/). The final dataset comprised 25 *in vivo* studies [11–35].

**Table-1:** Studies examining the effects of zinc nanoparticles and zinc conventional addition on broiler chicken were included in the meta-analysis.

No.	Authors	Dose of zinc addition (mg/kg)	Type of Zinc Source	n	Country	Strains of chicken	Observation period (days)
1	Ramiah *et al*. [11]	60–120	ZnO, ZnO NPs	240	USA	Male broiler chicks (Cobb 500)	42
2	Lukasiewicz *et al*. [12]	59–114	ZnO; ZnO NPs	308	Poland	Ross 308 chicks	42
3	Hidayat *et al*. [13]	40–220	ZnSO_4_; Nano Zinc Phytogenic	360	Indonesia	Broiler chickens (Ross 308)	33
4	Khah *et al*. [14]	36.29–156.29	ZnO; ZnO NPs	300	Iran	Male broiler chicks (300)	21
5	Alkhtib *et al*. [15]	50	methionine-Zinc chelate; Zinc NPs	384	United Kingdom	Ross 308 male broilers	35
6	El-Shenawy *et al*. [16]	50–100	ZnO; ZnO NPs	180	Egypt	Ross-308 broiler chickens	35
7	Eskandani *et al*. [17]	60–120	ZnSO_4_; Zn amino acid complex; Zinc NPs	240	Iran	Ross-308 mixed-sex broiler chicks	42
8	Yusof *et al*. [18]	100–200	ZnO; ZnO NPs	180	Malaysia	Broiler chicks (female; Cobb500 strain)	35
9	Qu *et al*. [19]	40	ZnO; ZnO NPs	75	China	Male Arbor Acres broiler chickens	42
10	Mozhiarasi *et al*. [20]	20–80	Zn organic; Zinc NPs	150	India	Broiler chicks (Cobb 400)	35
11	Mohammed *et al*. [21]	5–120	ZnO; ZnO NPs	360		Broiler Ross 308	42
12	Zhang *et al*. [22]	40.78–203.38	ZnSO_4_; ZnO NPs	320	China	Male Arbor Acres chicks	42
13	El-Maddawy *et al*. [23]	75–95	ZnO; ZnO NPs	160	Saudi arabia	Broiler chicks (Ross 308)	42
14	Sagar *et al*. [24]	40–80	Inorganic Zinc; Organic Zinc; Zinc NPs	384	India	CARIBRO-Vishal broiler chicks	42
15	Hafez *et al*. [25]	40–80	ZnO; ZnO NPs	90	Egypt	Ross 308 broiler chicks	14
16	Radi *et al*. [26]	40–3000	ZnO; ZnO NPs	102	Egypt	Hubbard broiler chicks	35
17	Dosoky *et al*. [27]	5–80	ZnO; ZnO NPs	224	Egypt	Male Cobb 500 chicks, white feather chicks	35
18	Zhao *et al*. [28]	20–100	ZnO; ZnO NPs	136	China	Male broilers (aged 6 days; Ross 308 strain)	42
19	Zhang *et al*. [29]	80–200.74	ZnSO4; ZnO NPs	250	China	Arbor Acres chicks (half male and half female)	21
20	Abdel-Wareth *et al*. [30]	76–136	ZnO; ZnO NPs	120	Egypt	Broiler chickens (Ross 308)	42
21	Alian *et al*. [31]	13.3–53.3	ZnO; Zn Lysine; ZnO NPs	156	Egypt	Ross 308 broiler chicks	35
22	Mozhiarasi *et al*. [32]	20–80	Inorganic zinc; Zinc Methionine; ZnO NPs		India	Broiler chicks (Cobb 400)	35
23	Hatab *et al*. [33]	22.1–82.1	ZnO; ZnO NPs	180	Egypt	Broiler chicks (Ross308)	38
24	Hatab *et al*. [34]	25–85	ZnO; ZnO NPs	120	Egypt	Broiler chicks (unsexed males and females, Cobb)	35
25	Lail *et al*. [35]	5–60	ZnO; ZnO NPs	150	Pakistan	Broiler chicks	35

NPs=Nanoparticles; ZNO=Zinc oxide

### Dataset characteristics

Table-1 shows that two Zn sources were used: Zn NPs and conventional Zn. The added doses of conventional Zn ranged from 0 to 3000 mg/kg feed, whereas the doses of Zn NPs ranged from 5 to 220 mg/kg. The additional Zn dose excluded the Zn derived from the feed. While tabulating the data into the dataset, similar variables were converted into the same measurement units to facilitate further analysis. The data entered into Microsoft Excel included information on the bibliography, Zn source, strains of broiler chickens, age of broiler chickens, and country. The response variables entered into the database comprised 11 groups. These groups included broiler chicken performance, with observations of average daily gain (ADG), average daily feed intake (ADFI), and feed conversion ratio (FCR). The parameters for carcass cuts and visceral organ weights included dressing, breast, thigh, abdominal fat, and gizzard and liver percentages. Lymphoid organ weight parameters included spleen, bursa, and thymus percentages.

Nutrient digestibility parameters included the percentages of dry matter, crude protein, ether extract, and crude fiber. The intestinal villus parameters included villus height (VH), crypt depth (CD), ratio of VH to CD, and villus width. The Zn, calcium, and phosphorous concentration parameters included observations of the Zn concentration in the muscle, liver, bone, and serum as well as the calcium and phosphorous concentrations in the serum. The hematological parameters included red blood cells (RBCs), white blood cells (WBCs), heterophil/lymphocyte (HL) ratio, hemoglobin (Hb), and packed cell volume (PCV)/hematocrit. Blood parameters included serum glucose, total protein, albumin, globulin, cholesterol, triglyceride, high-density lipoprotein (HDL), and low-density lipoprotein (LDL). The immunoglobulin (Ig) parameter group included total Ig, IgG, IgM, and sheep RBCs (SRBCs). The antioxidant activity parameters included observations of malondialdehyde (MDA), superoxide dismutase (SOD), catalase (CAT), alkaline phosphatase (ALP), aspartate aminotransferase (AST) in serum, and alanine aminotransferase (ALT) in serum. Finally, the intestinal bacterial population parameters included observations of *Escherichia coli* and *Salmonella* spp.

### Statistical analysis

The acquired data were analyzed using the analytical model proposed by St-Pierre [36], employing Statistical Analysis System software (SAS 9.3, https://welcome.oda.sas.com/) and mixed procedures (PROC MIXED). Statistical modeling determined the response variables affected by the addition of Zn (conventional Zn vs. Zn NPs) to broiler chickens. To test the effectiveness of the Zn form, we categorized it according to the form. The Zn forms used for comparison were conventional Zn and Zn NPs. As a result, two forms of Zn were used for the treatment. The following statistical model was used to analyze the Zn form categories:

Yij = μ + Si + τj + sτij + e ij

The expected outcome for dependent variable *Y* is denoted as Y_ij_, where μ represents the overall mean, S_i_ represents the random effect of the *i*^th^ study, and e_i_ represents the residual error. The significance of the effects was determined at a p = 0.05, or in cases where the value approached 0.05, a trend was observed.

## Results

Table-2 presents statistical descriptions of the variables and parameters used in this study. Conventional Zn doses range from 5 to 3000 mg/kg, whereas nano Zn particle doses range from 5 to 220 mg/kg. The data ranges for performance parameter groups are as follows: ADG ranged from 11.76 to 99.79 g/day/bird; ADFI ranged from 4.70 to 178.10 g/d/bird; and FCR ranged from 1.08 to 3.62. Carcass cuts and visceral organ weight parameter groups had the following data ranges: dressing percentage (60.83%–83.80%), breast (22.52%–34.66%), thigh (17.26%–31.32%), abdominal fat (0.41%–1.35%), gizzard (1.35%–2.75%), and liver (1.33%–3.14%). Meanwhile, the lymphoid organ weight parameter group had the following data ranges: spleen (0.07%–0.29%); bursa (0.06%–2.80%); thymus (0.18%–0.88%). The nutrient digestibility parameter group had the following data ranges: dry matter (70.20%–75.92%); crude protein (63.99%–94.08%); ether extract (64.36%–82.42%); crude fiber (21.21%–40.24%). The intestinal villi parameter group had the following data ranges: VH (114.10–1503 µm); CD (30.74–1062.32 µm); villi width (63.69–191 µm). The Zn, calcium, and phosphorus concentration parameter groups had the following data ranges: Zn in muscle (0.80–27.61 mg/kg); Zn in liver (2.40–531.30 mg/kg); Zn in bone (88.29–783.90 mg/kg); Zn in serum (2.09–204.39 ppm); calcium in serum (7.22–13.29 mg/dL); and phosphorus in serum (4.03–13.29 mg/dL).

**Table-2:** Summary of the statistical data of the included studies.

Parameters	Unit	Min.	Mean	Max.
Dose of conventional Zinc	mg/kg	5	242.76	3000
Dose of Zinc nanoparticles	mg/kg	5	79.44	220
Performance				
Average daily gain	g/d/bird	11.76	49.17	99.79
Average daily feed intake	g/d/bird	4.70	83.35	178.10
Feed conversion ratio		1.08	1.68	3.62
Carcass cuts and visceral organs weight percentage				
Dressing	%	60.83	73.70	83.80
Breast	%	22.52	27.83	34.66
Thigh	%	17.26	21.65	31.32
Abdominal fat	%	0.41	0.93	1.35
Gizzard	%	1.35	2.02	2.75
Liver	%	1.33	2.08	3.14
Lymphoid organ weight percentage				
Spleen	%	0.07	0.14	0.29
Bursa	%	0.06	0.23	2.80
Thymus	%	0.18	0.34	0.88
Nutrient digestibility				
Dry matter	%	70.20	73.07	75.92
Crude protein	%	63.99	84.87	94.08
Ether extract	%	64.36	77.01	82.42
Crude fiber	%	21.21	27.12	40.24
Intestinal villi				
Villus height	μm	114.10	741.09	1503.00
Crypt depth	μm	30.74	172.17	1062.32
Villus height/Crypt depth ratio		4.42	8.99	18.60
Villi width	μm	63.69	131.22	191.00
Mineral (Zn, Ca, and Phosphorous) concentration				
Zinc in muscle	mg/kg	0.80	5.45	27.61
Zinc in liver	mg/kg	2.40	49.22	531.30
Zinc in tibia	mg/kg	88.29	317.63	783.90
Zinc in serum	ppm	2.09	99.76	204.39
Calcium in serum	mg/dL	7.22	10.00	13.29
Phosphorus in serum	mg/dL	4.03	7.21	13.29

d=day, g=gram, kg=kilogram, log=logarithmic, mg=milligram, mL=Milliliter, ppm=parts per million

Table-3 displays the statistical description of hematological parameter groups with the following data ranges: RBC (1.17–4.09 × 10^3^/mm^3^); WBCs (14.42–240.35 × 10^3^/mm^3^); HL ratio (0.30–0.61); Hb (7.24%–12.53%); PCV/Hematocrit (21.89%–40.39%). Serum glucose (113.83–307 mg/dL), serum total protein (2.22–6.13 g/dL), serum albumin (1.31–3.50 g/dL), serum triglycerides (18.50–216.31 mg/dL), HDL (48.18–103 mg/dL), and LDL (81.89–420 mg/dL) are the data ranges for the blood parameter group. The following data ranges are part of the Ig parameter group: IgM (1.95–7.17 log2), IgG (1.12–9.92 log2), total Ig (3.12–4.75 log2), and SRBC (2.56–7.47 × 10^6^/mL). MDA (0.18–12.57 mg/kg meat); SOD (4.39–62.20 U/L); CAT (1.54–8.90 U/mL); ALP (44.18–116 U/L); AST in serum (24.22–511 U/L); ALT in serum (8.50–64.33 U/L) are among the data ranges for the Antioxidant activity parameter group. The following data ranges were part of the intestinal bacterial population parameter group: *Salmonella* spp. (1–3.60 log colony-forming unit [CFU]/g) and *E. coli* (1–4.23 log CFU/g).

**Table-3:** Summary of the statistical data of the included studies.

Parameters	Unit	Min.	Mean	Max.
Hematology				
Red blood cells	10^3^/mm^3^	1.17	2.44	4.09
White blood cells	1^0^3/mm^3^	14.42	54.49	240.35
Heterophile/Lymphocites ratio		0.30	0.40	0.61
Hemoglobin	%	7.24	11.01	12.53
Packed cell volume/hematocrit	%	21.89	34.82	40.39
Blood parameters				
Serum glucose	mg/dL	113.83	185.07	307.00
serum total protein	g/dL	2.33	3.79	6.13
Serum albumin	g/dL	1.31	2.18	3.50
Serum globulin	g/dL	1.34	2.02	3.03
Serum cholesterol	mg/dL	113.00	192.34	290.62
Serum triglycerides	mg/dL	18.50	149.51	216.31
High-density lipoprotein	mg/dL	48.18	70.94	103.00
Low-density lipoprotein	mg/dL	81.89	230.91	420.00
Immunoglobulin (Ig)				
Total Ig	Log2	3.12	4.12	4.75
IgG	Log2	1.12	4.21	9.92
IgM	Log2	1.95	3.68	7.17
Sheep red blood cells	10^6^/mL	2.56	5.61	7.47
Antioxidant activity				
Malondialdehyde	mg/kg meat	0.18	4.63	12.57
Superoxide dismutase	U/I	4.39	32.56	62.20
Catalase	U/mL	1.54	2.97	8.90
Alkaline phosphatase	U/I	44.18	528.01	1116.00
Aspartate aminotransferase in serum	U/I	24.22	140.97	511.00
Alanine aminotransferase in serum	U/I	8.50	31.89	64.33
Intestinal bacterial population				
*Escherichia coli*	log CFU/g	1.00	3.02	4.23
*Salmonella* spp.	log CFU/g	1.00	2.21	3.60

g=gram, kg=kilogram, log=logarithmic, mg=milligram, mL=Milliliter, mm=Millimeters

Table-4 presents the findings, which demonstrate that the average amount of conventional Zn used in this study was 24.76 mg/kg. Zn comparison, Zn NPs were administered at an average dose of 79.44 mg/kg. Despite the notable dosage discrepancies between the two forms of Zn, the performance outcomes remained consistent. Specifically, there were no significant differences in the ADG and ADFI between conventional Zn and Zn NPs (p > 0.05). However, the use of Zn NPs yielded a considerably improved FCR when juxtaposed with conventional Zn (1.70 vs. 1.79; p < 0.05). Examination of conventional Zn and Zn NPs for carcass cut and visceral organ percentage parameters revealed that the implementation of Zn NPs exhibited a notably advantageous effect (p < 0.05) on the dressing percentage parameter and significantly reduced the percentage of abdominal fat (p < 0.05). A decrease in abdominal fat indicated superior carcass quality and enhanced nutrient utilization efficiency, thereby underscoring the beneficial effects of Zn NPs. Moreover, at the lower dosage of Zn NPs employed in this study, no statistically significant differences (p > 0.05) were observed in the breast, thigh, gizzard, and liver tissues compared to conventional Zn.

**Table-4:** The effects of conventional zinc compared to zinc nanoparticles on production performance, carcass cuts and visceral organs percentage, lymphoid organ weight percentage, nutrient digestibility, intestinal villi, mineral (Zn, Ca, and P) concentration of broiler chicken.

Parameters	Unit	n	Type of zinc		p-value

			Conventional zinc	Zinc nanoparticles	
Dose average	mg/kg		242.76	79.44	
Performance					
Average daily gain	(g/d/bird)	280	48.52	51.32	0.224
Average daily feed intake	(g/d/bird)	249	81.07	83.53	0.624
Feed conversion ratio		271	1.79^a^	1.70^b^	0.015
Carcass cuts and visceral organs percentage					
Dressing percentage	%	47	72.35^a^	74.40^b^	<0.001
Breast	%	22	27.28	28.03	0.694
Thigh	%	16	20.69	21.06	0.897
Abdominal fat	%	11	1.08^a^	0.91^b^	0.040
Gizzard	%	23	2.11	2.03	0.632
Liver	%	44	2.07	2.12	0.531
Lymphoid organ weight percentage					
Spleen	%	50	0.11^a^	0.14^b^	0.007
Bursa	%	46	0.32	0.18	0.246
Thymus	%	42	0.34	0.37	0.154
Nutrient digestibility					
Dry matter	%	11	72.20	72.55	0.582
Crude protein	%	11	78.77	82.38	0.063
Ether extract	%	11	73.19	75.71	0.059
Crude fiber	%	11	26.51	29.86	0.059
Intestinal villi					
Villus height (VH)	μm	43	736.63	827.77	0.151
Crypt depth (CD)	μm	43	176.64	214.93	0.495
VH/CD ratio		40	7.2	8.03	0.205
Villi width	μm	11	129.81	137.24	0.471
Mineral (Zn, Ca, and P) concentration					
Zinc in muscle	mg/kg	56	7.62	8.01	0.320
Zinc in liver	mg/kg	70	62.58	68.78	0.586
Zinc in bone	mg/kg	11	282.1	360.00	0.221
Zinc in serum	ppm	19	90.69	98.05	0.217
Calcium in serum	mg/dL	23	10.18	10.00	0.378
Phosphorus in serum	mg/dL	23	7.73	7.54	0.291

^a,b^=Different superscripts in the same column indicate a significant difference (p < 0.05). Ca=Calcium, g=Gram, kg=Kilogram, log=Logarithmic, mg=Milligram, mL=Milliliter, ppm=Parts per million, P*=*Phosphorus

The comparison of utilizing conventional Zn with Zn NPs concerning the parameter of lymphoid organ weight percentage is as follows: The utilization of Zn NPs demonstrated a markedly superior effect (p < 0.05) on the dressing percentage and significantly elevated the percentage of spleen weight in comparison to the utilization of conventional Zn. Considering the lower dose of Zn NPs employed in this investigation, the use of Zn NPs also resulted in bursa and thymus percentages that were not significantly different (p > 0.05) from those of conventional Zn. Comparison of conventional Zn with Zn NPs with respect to the parameters of nutrient digestibility: On utilizing a lower dose of Zn NPs in this study, the use of Zn NPs yielded dry matter, crude protein, ether extract, and crude fiber digestibility that were not significantly different (p > 0.05) from the use of conventional Zn. Similarly, when comparing the utilization of conventional Zn with Zn NPs with the parameter group of intestinal villi, the following results were observed. With a lower dose of Zn NPs employed in this study, the utilization of Zn NPs yielded VH, CD, VH/CD ratio, and villus width, which were not significantly different (p > 0.05) compared with the use of conventional Zn. Meanwhile, the comparison of the utilization of conventional Zn with Zn NPs relating to the parameter groups of Zn, calcium, and phosphorus concentrations was as follows: When employing a lower dose of Zn NPs in this study, the concentrations of Zn in muscle, liver, tibia, and serum, as well as phosphorus in serum, did not show significant differences (p > 0.05) compared to the use of conventional Zn.

Table-5 compares the effects of traditional Zn usage and Zn NPs on various parameters. In the hematology parameters group, the comparison between conventional Zn usage and Zn NPs revealed that the utilization of Zn NPs led to a significant increase (p < 0.05) in Hb and PCV/hematocrit. Furthermore, Zn NPs also significantly decreased (p < 0.05) the HL ratio. When considering the lower dosage of Zn NPs employed in this study, RBC and WBCs were not significantly different (p > 0.05) from the conventional Zn usage. In the blood parameters group, the comparison between conventional Zn usage and Zn NPs revealed that the use of Zn NPs led to a significant increase (p < 0.05) in serum globulin compared with conventional Zn usage. In addition, at the lower dose of Zn NPs used in this study, serum glucose, serum total protein, serum albumin, serum cholesterol, serum triglycerides, HDL, and LDL levels were not significantly different (P > 0.05) from those of conventional Zn use. The effects of Zn NPs on Ig parameters, namely, total Ig, IgG, and IgM, were also not significantly different (p > 0.05). These effects were achieved at a lower Zn NP dose than with conventional Zn usage. The utilization of Zn NPs led to a significant increase (p < 0.05) in SRBC.

**Table-5:** The impact of conventional zinc compared to zinc nanoparticles on the hematology, blood parameters, immunoglobulin, antioxidant activity, and intestinal bacterial population of broiler chickens.

Parameters	Unit	n	Type of zinc		p-value

			Zinc conventional	Zinc nanoparticles	
Dose average	mg/kg		242.76	79.44	
Hematology					
Red blood cells	10^3^/mm^3^	23	2.49	2.78	0.057
White blood cells	10^3^/mm^3^	19	73.30	74.30	0.224
HL ratio		31	0.47^a^	0.42^b^	0.001
Hemoglobin	%	20	10.36^a^	11.37^b^	0.028
Packed cell volume/Hematocrit	%	20	32.74^a^	35.89^b^	0.032
Blood parameters					
Serum glucose	mg/dL	25	182.55	179.91	0.740
Serum total protein	g/dL	42	3.73	3.79	0.246
Serum albumin	g/dL	37	2.21	2.20	0.654
Serum globulin	g/dL	26	1.88^a^	2.09^b^	0.001
Serum cholesterol	mg/dL	36	194.95	187.44	0.235
Serum triglycerides	mg/dL	32	151.54	150.21	0.668
High-density lipoprotein	mg/dL	20	66.13	67.27	0.425
Low-density lipoprotein	mg/dL	20	201.85	195.71	0.356
Immunoglobulin (Ig)					
Total Ig	Log2	12	4.02	4.17	0.670
IgG	Log2	27	4.95	4.81	0.154
IgM	Log2	27	4.02	4.07	0.863
Sheep red blood cells	10^6^/mL	30	5.41^a^	6.17^b^	0.048
Antioxidant activity					
Malondialdehyde	mg/kg meat	121	4.27	3.91	0.403
Superoxide dismutase	U/L	76	24.32^a^	29.31^b^	0.009
Catalase	U/mL	32	2.82	3.02	0.791
Alkaline phosphatase	U/L	20	433.3^a^	413.57^b^	0.003
Aspartate aminotransferase in serum	U/L	39	127.91	118.29	0.105
Alanine aminotransferase in serum	U/L	49	31.13	30.31	0.318
Intestinal bacterial population					
*Escherichia coli*	log CFU/g	12	3.73	2.66	0.065
*Salmonella* spp.	log CFU/g	12	2.75^a^	1.93^b^	<0.001

^a,b^=Different superscripts in the same column indicate a significant difference (p < 0.05). CFU=Colony forming unit, g=Gram, kg=Kilogram, log=Logarithmic, mg=Milligram, mL=Milliliter

A comparison of conventional Zn usage with Zn NPs regarding the antioxidant activity parameter group is as follows: The use of Zn NPs significantly (p < 0.05) increased SOD activity but decreased ALP activity. At the lower dosage of Zn NPs utilized in this particular investigation, the use of Zn NPs also led to the production of MDA, CAT, AST, and ALT, which did not exhibit any significant differences (p > 0.05) when compared to the conventional utilization of Zn. In comparison with the conventional usage of Zn, the utilization of Zn NPs significantly reduced the population of intestinal bacteria, specifically *E. coli* and *Salmonella* spp., with a lower dosage of Zn NPs used in this study.

## Discussion

This meta-analysis compared the effects of different types of Zn, namely, conventional Zn and Zn NPs. In this study, the mean doses of conventional Zn and Zn NPs differ significantly, namely, 242.76 versus 79.44 mg/kg, respectively. Despite the substantial differences in doses, this study showed that Zn NPs often exert biological effects on broiler chickens that are not different from those of conventional Zn. In fact, for certain parameters, the effects of Zn NPs appear to be even better. This indicates that reducing the size of the Zn particles to NPs improves the efficiency of Zn utilization based on the observed parameters.

In this study, the effects of using Zn NPs on broiler performance parameters (Table-4) indicated that at a much lower dose, Zn NPs can produce effects that are not significantly different from conventional Zn for parameters such as ADG and ADFI. Zn NPs have better effects on FCR than conventional Zn. This is good news because using Zn NPs can reduce the Zn content in the feed, resulting in better Zn utilization efficiency. The excretion of Zn through feces due to the use of high-dose Zn in feed is indeed an important issue, as it has been reported to cause environmental pollution [37]. Therefore, Zn NPs are a suitable solution for reducing environmental pollution caused by contaminated Zn discharged through feces [38]. Animal excrement is a significant contributor of heavy metals in natural environments [39]. High Zn levels in the water and soil can reduce crop yield [40]. This is primarily because Zn elevates the acidity of water, disrupting the functioning of the soil, and impeding the activities of microorganisms and earthworms [41]. Consequently, the decomposition of organic matter was slowed [42]. Researchers have devised Zn NPs to mitigate the environmental pollution caused by Zn discharged through fecal matter [43, 44]. The primary objective of developing Zn NPs is to lower the dosage of Zn in the diet [45]. By reducing the amount of Zn added to the diet, there will consequently be a reduction in the amount of Zn excreted in the feces. Zn NPs exhibited greater efficacy than larger Zn particles at lower doses [46].

This study also revealed that Zn NPs positively affected dressing percentage, carcass cuts, and visceral organs (Table-4). This can be seen from the higher dressing percentage produced by Zn NPs compared to conventional Zn (Table-4). Zn NPs also improved carcass quality, as evidenced by a decrease in abdominal fat content. Abdominal fat is considered ineffective for nutrient utilization because it is not transformed into meat [47]. Reducing fat accumulation in the body parts of chickens has become a significant focal point in broiler research. This development is driven by consumer demand for superior-quality poultry products that satisfy stringent health standards. Excessive fat content in animal products is a fundamental catalyst of obesity and coronary heart disease [48]. Reducing abdominal fat in broilers not only decreases production expenses, but also enhances feed efficiency because abdominal fat is perceived as waste [49]. Incorporating Zn NPs into broiler diets enhances carcass quality by diminishing the proportion of abdominal fat [50, 51].

At a lower dose, Zn NPs maintained nutrient digestibility, which was not significantly different from the nutrient digestibility in broiler chickens administered with conventional Zn at a much higher dose. The manifestation of Zn utilization efficacy in the form of NPs was also apparent in the parameters of the intestinal villi, wherein the dimensions of VH, CD, VH/CD ratio, and villus width exhibited comparable magnitudes, regardless of whether Zn NPs or conventional Zn was used. The advantage of employing Zn NPs was that the administered quantity was significantly lower than that of conventional Zn within the confines of this investigation (79.44 vs. 242.76 mg/kg).

The effects of Zn NPs on production performance, carcass percentage, visceral organs, nutrient digestibility, and intestinal villi size in broiler chickens when using Zn NPs are closely related for several reasons. Zn in NP form has vastly different physical properties from its conventional counterparts, thus contributing to various new applications [52]. Because of their significantly reduced particle size, they exhibit new and improved physical, chemical, and biological activities that differ from those of conventional particles [53]. NPs exhibit higher absorption rates in chicken bodies [54]. The functioning of NPs relies on several factors, including particle size [55]. Particles smaller than 300 nm can disperse within the bloodstream, whereas those smaller than 100 nm can reach various tissues [56]. Zn NPs can permeate the small intestine and form mucosal barriers within the body [57]. In this context, the absorption of NPs has been reported to be 15–250 times higher than that of conventional Zn [58]. The increased absorption of Zn by the NPs can be attributed to the larger surface area of the NPs, which facilitates better interactions [59]. This, in turn, leads to a prolonged residence time in the intestine and a reduction in intestinal clearance mechanisms. Moreover, NPs enhance tissue penetration and penetration into the epithelial layer, resulting in more efficient cell absorption [60].

The increased surface area of NPs facilitates better biological interactions, prolongs intestinal retention time, and enhances bioavailability and functionality [61]. Due to their small size, NP absorption by the epithelium of the small intestine is much easier [62]. The absorption of NPs through the mucosal layer depends on the charge on the surface and pH of the surrounding environment. Changes in pH alter the surface charge, causing agglomeration and size changes [63]. Zn NPs are absorbed through the villus epithelium into the bloodstream and transported to the liver and spleen. Zn NPs can penetrate various organs, such as the bloodstream, brain, lungs, heart, kidneys, spleen, liver, intestines, and stomach, by traversing the small intestine [64]. Nevertheless, the extent of absorption in both the intestinal epithelium and other body tissues is heavily influenced by particle size. For Zn molecules to swiftly traverse the stomach wall and gain entry into cells faster than larger Zn particles, the size of the Zn NPs must be restricted to <100 nm [65].

Zn NPs enhanced the weight of lymphoid organs, specifically the spleen, compared to the traditional Zn group. These observations indicate that the utilization of Zn NPs positively affects the overall health of broiler chickens, as an increase in lymphoid organ weight indicates a more active and improved immune response [66]. Zn plays a critical role in the immune system of broilers. Lymphoid organs are integral to the structure and function of the immune system in broilers and protect the body against microbial attack. Zn is widely recognized for its indispensable role in the functioning, structure, and development of the animal immune system [67]. Zn is necessary for lymphocyte proliferation. The presence of Zn in poultry feed directly influences the size of lymphoid organs, which is closely associated with the functionality of T cells and decreases when the diet is supplemented with insufficient levels of Zn [68]. The size of a splenic mass is closely associated with humoral immunity. A larger spleen results in heightened humoral immunity; conversely, a smaller spleen results in reduced humoral immunity [69].

The use of Zn NPs demonstrated enhanced efficacy in the deposition of Zn, Ca, and phosphorus in meat and bones (Table-4). This was attributed to the capability of the Zn NPs to generate Zn concentrations that were not significantly different from those of traditional Zn, albeit at considerably lower doses. The superior efficiency of Zn deposition resulting from Zn NPs can be linked to the nanoparticulate form of Zn, which exhibits greater bioavailability than conventional Zn. The enhanced bioavailability of Zn NPs can modify the deposition of minerals because of their increased interactions with biological tissues [38]. This can be attributed to the activity of metallothionein, a cysteine-rich protein that binds divalent cations and regulates the pool and turnover of trace elements [3]. An alternative theory supporting these findings suggests that Zn NPs can permeate the liver cells through the bloodstream or interstitial spaces. The reduced size of NPs facilitates greater uptake and interaction with biological tissues because NPs occupy transitional zones between individual atoms or molecules and the bulk material [26]. Serum Zn concentration can serve as an indicator of the quantity of Zn assimilated by poultry during a specific time frame [70].

Consequently, serum Zn concentration is widely used to gauge Zn status, with low values indicating early-stage Zn deficiency. Bones function as mineral reservoirs. Zn is utilized by the body during Zn deficiency [71]. Hence, the Zn content in bones is a reliable measure of the biological availability of Zn in poultry diets. Adequate Zn concentrations are essential for bone growth, development, and mineralization [72]. Bones serve as storage sites for Zn within the body and are a reserve that is trapped during Zn deficiency. Zn metabolism in bones is highly dynamic, with Zn redistributed from the bones to other tissues when the body is deficient in Zn. The mineral content of tissues is frequently used as an indicator of the mineral status of animals and the level of mineral intake by animals [73].

The use of Zn NPs in this study had positive effects on the health of broiler chickens, as observed in the hematological parameters (Table-5). The use of Zn NPs increased Hb and PCV/hematocrit levels compared to conventional Zn. Zn NPs also decreased the HL ratio compared with conventional Zn. A decreased HL ratio in poultry can be regarded as a reliable indicator of the overall well-being and immune system function. The HL ratio is the ratio of the number of heterophilic cells (a variety of WBCs implicated in nonspecific immune responses) to the number of lymphocytes (a variety of WBCs implicated in specific immune responses) [74]. When poultry experiences stress or infection, there is typically an increase in the number of heterophilic cells and a reduction in the number of lymphocytes, resulting in an increased HL ratio. This is frequently viewed as an indication that poultry are stressed or exposed to pathogens, which may imply to reduce well-being and immune system disruption [75]. The use of Zn NPs to diminish the HL ratio suggests that Zn NPs exert a superior effect compared with traditional Zn. Adequate Zn levels are necessary for normal lymphocyte production during development [76]. Consequently, a shortage of Zn decreases the numbers of peripheral T cells, helper T cells, and thymus cells. Incorporation of Zn facilitates optimal lymphocyte development and alleviates stress. The increase in the number of lymphocytes and the decline in the H/L ratio were likely attributable to a decrease in glucocorticoid secretion [77].

Hemoglobin (Hb), a protein found in RBCs, is vital for transporting oxygen throughout the body, specifically from the lungs. Consequently, elevated Hb levels can enhance the capacity of the body to deliver oxygen to various tissues, such as muscles and essential organs. High Hb levels indicate good health in chickens [78]. Zn NPs can increase Hb levels, indicating that Zn NPs have a better effect than conventional Zn [79]. An increase in PCV or hematocrit levels in chickens is considered a good indicator of health. The PCV quantifies the proportionate volume of erythrocytes in avian blood, and an elevation in the PCV generally indicates an increase in the number of erythrocytes. This indicates that chickens have a better capacity to carry oxygen throughout their body, which is important for optimal health and performance. The use of Zn NPs can increase the PCV, indicating that Zn NPs have a better effect than conventional Zn [68, 80]. Zn NPs can increase serum globulin levels (Table-5) compared to conventional Zn. Globulin is a blood protein produced by the liver that plays a crucial role in the immune system of the body. Increased serum globulin levels indicated that the chicken immune system was active [81]. A positive sign indicated an increase in serum globulin levels in the context of a normal immune response. Other blood parameters, such as serum glucose, serum total protein, serum albumin, serum cholesterol, serum triglycerides, HDL, and LDL, showed no discernible disparity between the Zn NP and conventional Zn groups [82].

Nevertheless, these achievements were attained using conventional Zn at significantly higher concentrations than with Zn NPs, suggesting that the utilization of Zn NPs is more effective in terms of Zn assimilation when considering their impact on hematological parameters. Zn is intrinsically interconnected with blood protein levels due to its association with the metabolic functionality of nutrients in the body. Zn facilitates enzymatic and physiological processes within the body, including nutrient digestion in the gastrointestinal tract [83]. Furthermore, blood protein levels are intricately linked to the duration of nutrient digestion in the gastrointestinal tract, and Zn plays a crucial role in nutrient digestion [38]. The health status of an individual can be evaluated from various perspectives, including protein content. Plasma proteins, including albumin, globulin, and fibrinogen, play a pivotal role in maintaining osmotic pressure, serving as a source of amino acids for tissues, facilitating the transportation of nutrients to cells, eliminating waste products to secretory organs, and sustaining acid-base equilibrium (buffer) [23].

Ig parameters, including total Ig, IgG, and IgM, did not differ between the Zn NPs and conventional Zn groups. However, these achievements were attained with the use of conventional Zn at much higher concentrations than with Zn NPs, indicating that the use of Zn NPs appears to be more efficient in Zn utilization when considering their effects on Igs. IgG is a protein produced by the immune system that protects against infections and diseases [25]. The total IgG, IgG, and IgM levels in broiler chickens have important functions. Total Ig encompasses all types of Igs produced by the chicken body [19]. The main function of total Ig is to protect the body from infections and diseases by recognizing and binding to pathogens such as bacteria, viruses, and fungi [31]. High total Ig levels indicate that the immune system is actively fighting infections and other disease conditions [80]. IgG is the predominant form of Ig in the bloodstream of broilers. The primary function of IgG is to protect against bacterial and viral infections. IgG also provides passive protection to chicks through transfer from the mother to the egg (maternal immunity) [82]. IgM is the first type of Ig that responds to new infections [31]. IgM contributes to the fight against early infections by activating the immune system to combat pathogens. Overall, total Ig, IgG, and IgM levels in broiler chickens are important indicators of the health and strength of the immune system [19]. The use of Zn NPs increased the number of SRBC compared with conventional Zn. SRBCs indicate the immune activity of chickens against specific antigens. An increase in SRBC values indicated that the chicken immune system responded more strongly to this antigen. Augmented SRBC values can be interpreted as an enhanced immune response, which may indicate a more robust state of the immune system and the body’s capacity to combat specific infections or ailments [84].

The use of Zn NPs had a greater effect on the antioxidant activity of broiler chickens than conventional Zn, as evidenced by increased SOD and decreased ALP levels (Table-5). The increased activity of SOD in broiler chickens is considered a positive response to oxidative stress. SOD is an antioxidant enzyme that plays a role in combating free radicals, particularly superoxide, a highly reactive free radical that can damage body cells [46]. When chickens experience oxidative stress, which may arise from various factors, such as heat stress, infection, or exposure to toxic substances, their bodies respond by augmenting the synthesis of antioxidant enzymes, such as SOD, to protect cells from oxidative damage [8]. Increased SOD activity in broiler chickens can help diminish cellular damage, repair DNA, and fortify the immune system, thereby enhancing overall health and performance [23]. Zn serves as a coenzyme for SOD and plays a significant role in the antioxidant system by functioning as an inhibitor of oxidative processes, thereby safeguarding proteins and enzymes and impeding the formation of free radicals [38]. SODs are ubiquitously distributed and offer protection against peroxidation in various tissues and organs. Zn is a vital constituent of Cu/Zn SODs, and SODs serve as a defense mechanism against oxidative stress at the cellular level, explaining why Zn deficiency can lead to increased production of free radicals [77]. Zn can augment immunity as it is an integral part of the cellular integration processes that participate in immune responses [13]. The pivotal role of Zn in the immune response is related to its influence on antioxidant defense mechanisms [45]. Elevated ALP activity in broilers is a major health concern. ALP is present in numerous body tissues, including the liver, bones, and intestines, and its levels can increase in response to specific health conditions [38]. Increased ALP levels are associated with liver, bone, and intestinal problems as well as oxidative stress. Elevated ALP levels are typically observed in patients with liver diseases, hepatitis, or cholestasis (bile duct obstruction). Similarly, bone diseases such as abnormal bone growth and bone damage can increase ALP levels. Conditions such as inflammatory bowel disease and intestinal obstruction can also increase ALP levels. Elevated ALP levels can also be a response to oxidative stress or general cell damage [77].

The Zn NPs exhibited better antibacterial activity than conventional Zn, as evidenced by the intestinal bacterial population (Table 5). The populations of *E. coli* and *Salmonella* spp. bacteria in the intestines of broiler chickens diminish when Zn NPs are used [85]. Some pathogenic strains of *E. coli* and *Salmonella* spp. can cause diseases in chickens or can be transmitted to humans via consumption of contaminated poultry products [86]. Therefore, it is important to reduce the number and types of pathogenic bacteria in chicken intestines and implement appropriate control measures to prevent the spread of infection, both in the chickens themselves and in the products produced [85]. The bactericidal action of Zn NPs occurs through the formation of reactive oxygen species (ROS) within bacterial cells (such as hydroxyl radicals, hydroperoxides, and superoxides), which can damage bacterial cell membranes and organelles, or Zn NPs alter the permeability of bacteria after the nanomines enter the cell plasma membrane, resulting in cell death [87]. The antimicrobial mechanism of action of Zn NPs involves microorganisms with a negative electromagnetic charge being attracted to metal oxides with a positive electromagnetic charge, causing oxidation and microbial death. The antibacterial activity of Zn NPs and other minerals depends greatly on their size, with smaller particles exhibiting better antimicrobial activity [9].

## Conclusion

This study identified a favorable effect of Zn NPs administered to broiler chickens instead of conventional Zn. Across the parameters evaluated in this study, including production performance; carcass cuts; visceral organ weight; lymphoid organ weight; nutrient digestibility; intestinal villi; mineral Zn, calcium, and phosphorus concentrations; hematology; blood parameters; Ig; and intestinal bacterial population, the use of Zn NPs was more efficient for Zn utilization than conventional Zn. Furthermore, the average dose of Zn NPs was much lower than that of conventional Zn (79.44 vs. 242.76 mg/kg), yet they provided similar or even superior effects compared to conventional Zn use. A limitation of this study is that the Zn NPs used were sourced from inorganic Zn NPs. Therefore, future research should focus on evaluating the efficiency of organic Zn NPs in broiler chicken feed.

## Authors’ Contributions

CH: Conception and design of the study, writing – original draft, statistical analysis, software, methodology, and investigation. SS: Software, data curation, and writing – review and editing. DNA: Methodology, conceptualization. validation, and software. RKR: Data curation, investigation, methodology, and writing – review and editing. BB: Conceptualization, validation, data curation, and investigation. SPG: Investigation, methodology, validation, and data curation. SAS: Writing – review and editing, conceptualization, validation, and data curation. BB: Methodology, validation, data curation, and investigation. AD: Writing – original draft, statistical analysis, data curation, and investigation. HZ: Writing – review and editing, methodology, validation, and data curation. AF: Conception and design of the study, validation, and data curation. SR: Software, methodology, validation, and data curation. IH: Validation, data curation, investigation, and methodology. ES: Conceptualization, investigation, methodology, and writing – review and editing. YRY: Conception and design of the study, conceptualization, validation, and software. AJ: Writing – review and editing, conception and design of the study, validation, and data curation. All authors have read, reviewed, and approved the final manuscript.
